# Mutations in fetal genes involved in innate immunity and host defense against microbes increase risk of preterm premature rupture of membranes (PPROM)

**DOI:** 10.1002/mgg3.330

**Published:** 2017-08-23

**Authors:** Bhavi P. Modi, Maria E. Teves, Laurel N. Pearson, Hardik I. Parikh, Hannah Haymond‐Thornburg, John L. Tucker, Piya Chaemsaithong, Nardhy Gomez‐Lopez, Timothy P. York, Roberto Romero, Jerome F. Strauss

**Affiliations:** ^1^ Department of Human and Molecular Genetics Virginia Commonwealth University Richmond Virginia; ^2^ Department of Obstetrics and Gynecology Virginia Commonwealth University Richmond Virginia; ^3^ Department of Anthropology Pennsylvania State University University Park Pennsylvania; ^4^ Department of Microbiology and Immunology Virginia Commonwealth University Richmond Virginia; ^5^ Perinatology Research Branch Eunice Kennedy Shriver National Institute for Child Health and Human Development NIH Detroit Michigan; ^6^ Department of Obstetrics and Gynecology University of Michigan Ann Arbor Michigan; ^7^ Department of Epidemiology and Biostatistics Michigan State University East Lansing Michigan; ^8^ Center for Molecular Medicine and Genetics Wayne State University Detroit Michigan

**Keywords:** Antimicrobial peptides, chorioamnionitis, defensins, fucosyltransferase, inflammasome, innate immunity, mannose‐binding lectin protein, preterm birth, preterm premature rupture of membranes

## Abstract

**Background:**

Twin studies have revealed a significant contribution of the fetal genome to risk of preterm birth. Preterm premature rupture of membranes (PPROM) is the leading identifiable cause of preterm delivery. Infection and inflammation of the fetal membranes is commonly found associated with PPROM.

**Methods:**

We carried out whole exome sequencing (WES) of genomic DNA from neonates born of African‐American mothers whose pregnancies were complicated by PPROM (76) or were normal term pregnancies (*N* = 43) to identify mutations in 35 candidate genes involved in innate immunity and host defenses against microbes. Targeted genotyping of mutations in the candidates discovered by WES was conducted on an additional 188 PPROM cases and 175 controls.

**Results:**

We identified rare heterozygous nonsense and frameshift mutations in several of the candidate genes, including *CARD6, CARD8, DEFB1, FUT2, MBL2, NLP10, NLRP12,* and *NOD2*. We discovered that some mutations (*CARD6, DEFB1, FUT2, MBL2, NLRP10, NOD2*) were present only in PPROM cases.

**Conclusions:**

We conclude that rare damaging mutations in innate immunity and host defense genes, the majority being heterozygous, are more frequent in neonates born of pregnancies complicated by PPROM. These findings suggest that the risk of preterm birth in African‐Americans may be conferred by mutations in multiple genes encoding proteins involved in dampening the innate immune response or protecting the host against microbial infection and microbial products.

## Introduction

Preterm birth, especially among African‐Americans, has challenged the U.S. health care system for decades (Kempe et al. 1992; Aveyard et al. 2002; Ahern et al. 2003; Behrman and Bulter 2007; Shen et al. 2008). The disparities in prematurity among U.S. populations is thought to be the result of multiple biological and environmental factors (Meis et al. 2000; Moutquin 2003; Anum et al. 2009b). Preterm premature rupture of membranes (PPROM) is the leading identifiable cause of preterm birth, and more common among African‐Americans. Our research has been focused on understanding the pathophysiology of PPROM, and the factors that contribute to population‐specific risk (Parry and Strauss 1998; Strauss 2013).

The notion that heritable factors play an important role in preterm birth is supported by studies based on twins (Boyd et al. 2009; Svensson et al. 2009; York et al. 2009, 2010, 2013, 2014, 2015). These studies demonstrated that both the fetal and maternal genomes contribute to the timing of parturition. In addition, there is increasing evidence that gene‐environment interactions amplify the effect of specific alleles (Wang et al. 2002; Macones et al. 2004; Anum et al. 2009a,2009b). However, the search for maternal and fetal genes linked to preterm birth has yet to produce robust and reproducible candidates. Although association studies have found significant relationships for some candidate genes, the primary reports and available meta‐analyses indicate that these associations are weak or population specific (e.g., Genc et al. 2002; Fujimoto et al. 2002; Ferrand et al. 2002b; Lorenz et al. 2002; Moore et al. 2004; Roberts et al. 1999; Romero et al. 2010; Simhan et al. 2003; Witkin et al. 2003; Wang et al. 2004, 2006, 2008; see Sheikh et al. 2016 for a recent review). Moreover, attempts to identify loci contributing to prematurity through genome‐wide association studies (GWAS) have not delivered strong candidates (Parets et al. 2015), prompting investigators to pursue alternative approaches to identify genes contributing to preterm birth (Bacelis et al. 2016; Brubaker et al. 2016). Recently, we took a different approach based on the hypothesis that rare mutations or damaging variants in multiple genes (which might escape detection by GWAS or standard association studies, especially with small sample sizes) make significant contributions to PPROM (Modi et al. 2017). The approach was based on mutation/damaging variant detection using whole exome sequencing (WES), which we applied in this study to explore fetal gene mutations in the innate immune system and PPROM.

Innate immunity encompasses recognition systems that detect molecules derived from bacteria and viruses (Pathogen‐Associated Molecular Patterns [PAMPs]) and endogenous alarmins (Damaged‐Associated Molecular Patterns [DAMPs]). Pattern recognition receptors (PRRs) responsible for the initiation of innate immune response induced by PAMPs and DAMPs include NOD‐like receptor family pyrin domain containing proteins and toll‐like receptors (TLR).

The response triggered by the PRRs includes activation of transcription of genes that encode cytokines and factors that resolve infection/inflammation (Brubaker et al. 2015). Enhanced production of pro‐inflammatory cytokines has been postulated to play a central role in preterm birth and PPROM (Parry and Strauss 1998; Murtha and Menon 2015; Gomez‐Lopez et al. 2017a,2017b; Romero et al., 2016). The pro‐inflammatory cytokines induce expression of matrix metalloproteinases which degrade fetal membrane extracellular matrix leading to rupture (Parry and Strauss 1998; Strauss 2013).

The innate immune system is modulated by a number of molecules that dampen/inhibit the inflammatory response triggered by “activating” toll‐like receptors and inflammasomes. Bacterial lipids and proteins derived from Gram negative and Gram positive bacteria (PAMPs) reaching the fetal membranes are potent activators of the innate immune response leading to inflammation. Numerous animal studies have shown that Gram negative bacterial lipopolysaccharide (LPS) precipitates preterm birth, and that the fetal membranes possess molecules that recognize bacterial products and trigger an inflammatory response, usually involving the activation of the transcription factor, NFkB (Courtois 2005). Endogenous enzymes (e.g., acyloxyacyl hydrolase, alkaline phosphatase) protect the host from the potent actions of LPS by altering LPS structure.

A number of endogenous proteins with antimicrobial activity like lactoferrin, mannose‐binding lectin 2, and fucosyltransferase 2 help protect exposed surfaces including mucosa, and the fetal membranes. The *FUT2 (OMIM:* (**+**182100) and *MBL2 (OMIM:* * 154545) genes are both expressed in the fetal membranes. The defensin family of genes expressed maternally and by the fetus probably combat bacteria ascending from the vagina, but possibly from other sources. Several defensins are known to be produced by fetal membranes including *DEFB1* (Avila 2016).

We analyzed WES data from neonatal DNA from 76 PPROM cases and 43 term controls born of African‐American mothers to identify damaging mutations in innate immunity genes and discovered that there was an overrepresentation of these damaging alleles in PPROM cases.

## Materials and Methods

### Study population

WES was performed on 76 PPROM cases and 43 healthy term control neonatal DNA samples all obtained in Richmond, Virginia. Additional genotyping of select variants was performed on an independent cohort of 188 case and 175 control fetal/neonatal DNA samples collected in Richmond, Virginia and Detroit, Michigan. DNA was isolated from cord blood or umbilical cords. Subjects were self‐reported African‐American women and their neonates receiving obstetrical care at MCV Hospitals, Richmond, VA (all samples in the initial WES) and Hutzel Hospital in Detroit, MI. The study was approved by the Institutional Review Boards of MCV Hospitals, Richmond, VA (IRB Number: HM15009); Wayne State University (IRB Numbers: 103897MP2F (5R), 082403MP2F (5R), 110605MP4F, 103108MP2F, 052308MP2F) as well as NICHD (National Institute of Child Health and Human Development) (IRB Numbers: 0H97‐CH‐N065, OH98‐CH‐N001, OH97‐CH‐N067, OH99‐CH‐N056, OH09‐CH‐N014). Subjects from Hutzel Hospital, Detroit, MI were enrolled under both Wayne State University as well as NICHD protocols and thus respective IRB numbers for both institutes are provided. Written informed consent was obtained from mothers before sample collection. Demographic and clinical data were obtained from surveys and medical records. Control DNA samples (*N* = 43 + 175) were obtained from neonates of singleton pregnancies delivered at term (>37 weeks of gestation) of mothers with no prior history of PPROM or preterm labor. Cases of PPROM (*N* = 76 + 188) were defined as neonates from pregnancies complicated by spontaneous rupture of membranes prior to 37 weeks of gestation. The diagnosis of membrane rupture was based on pooling of amniotic fluid in the vagina, amniotic fluid ferning patterns and a positive nitrazine test. Women with multiple gestations, fetal anomalies, trauma, connective tissue diseases, and medical complications of pregnancy requiring induction of labor were excluded. A DNA biobank at Virginia Commonwealth University and Hutzel Hospital of PPROM cases and term controls collected using the same criteria as those used for the WES cohort was employed for subsequent genotyping of selected mutations identified by WES (Modi et al. 2017).

### Ancestry estimates

Genetic ancestry was estimated to investigate population structure in the cases and control cohorts (Collins‐Schramm et al., 2003). Genetic ancestry estimates were generated in a two‐way model of admixture, European and West African, for the neonates of each self‐reported African‐American study subject using 102 ancestry informative markers (AIMs), single nucleotide polymorphisms with large allele frequency differences between ancestral populations, (Modi et al. 2017). The mean allele frequency difference between ancestral populations for the AIMs panel was delta (*δ*) = 0.733. The AIMs panel was derived from the overlap of the WES and the Illumina African American Admixture Mapping Panel (Illumina, San Diego, CA, USA) and genotyped using a custom iPLEX assay (Agena Biosciences, San Diego, CA, USA) for study subjects who were not part of the WES discovery set (Modi et al. 2017). Prior allele frequencies derived from the HapMap West Africans (YRI, Yoruba in Ibadan, Nigeria) and Europeans (CEU, CEPH Utah residents with ancestry from northern and western Europe) were used to estimate individual genetic ancestry following a maximum‐likelihood approach.

### Whole exome sequencing analysis

Whole exome capture and sequencing was performed at BGI (BGI, Cambridge, MA) using the SureSelect Target Enrichment System Capture Process followed by high‐throughput sequencing on an Illimina HiSeq2000 platform with 50–100× coverage. The bioinformatics analysis for variant discovery and annotation was performed as described earlier (Modi et al. 2017). In brief, sequences were mapped to the human reference genome (build hg19) using BWA, followed by marking PCR duplicates using Picard tools and base quality recalibration using GATK (Modi et al. 2017) GATK‐HaplotypeCaller was used to identify variants in individual samples, followed by joint genotyping of all samples in the cohort for population‐level analysis. The raw SNPs and INDELs were filtered for high quality and annotated for their functional effects using SnpEff tool and known variant databases like dbSNP, ClinVar, and the 1000 Genomes Project. Damaging missense variants were selected on the basis of most deleterious predictions in both Polyphen2 (HumDiv ‐ probably damaging) as well as SIFT (damaging) platforms. PCR and Sanger sequencing was used to validate mutations detected by WES (Table S1) or mutations were confirmed by custom genotyping.

### Custom genotyping

The variants identified and selected for further analysis from Whole Exome Sequencing were validated, and additional samples (an independent cohort of additional 188 cases and 175 controls) were genotyped for the selected variants. Genotyping was performed on the Agena (previously Sequenom) MassArray iPLEX platform following manufacturer's instructions at the University of Minnesota Genomics Center (Modi et al. 2017). The primer sets used for iPlex genotyping are presented in Table S2.

### Statistical analysis

Mean levels of demographic variables were tested using a 2‐tailed Student's *t*‐test. Count data (for gravidity and parity) was square root transformed before performing tests. *P*‐values <0.05 were considered statistically significant. The paired Wilcoxon rank‐sum test was used to assess significant differences in minor allele frequencies.

## Results

WES was performed on 76 PPROM and 43 healthy term control neonatal DNA samples. The demographic characteristics of the WES study population is presented in Table 1. The characteristics of the follow‐up cohort have been previously reported (Modi et al. 2017). With 152 chromosomes, the probability of detecting a variant with an allele frequency of 0.005 was 78%.

**Table 1:** Study subject characteristics for WES

Characteristic	Cases mean (SD)	Controls mean (SD)	*P*‐value
Maternal age (years)	27.18 (5.33)	26.02 (5.32)	0.256
Gestational age at delivery (weeks)	30.05 (4.17)	38.93 (1.16)	<0.001
Neonatal weight (kgs)	1.69 (1.59)	3.14 (0.46)	<0.001
Gravidity	3.53 (2.04)	3.25 (2.57)	0.555
Parity	1.47 (1.57)	1.35 (1.41)	0.657

PPROM cases, *N* = 76; Term controls, *N* = 43.

The WES PPROM cases and term controls had similar West African and European ancestry based on genotyping of 102 ancestry informative markers (Means ± SD; West African ancestry: PPROM cases: 0.695 ± 0.073 (mean ± SD); Term controls 0.698 ± 0.087 [*P* > 0.10]).

A total of 35 candidate genes were selected for investigation of nonsense mutations and insertions/deletions causing damaging frameshift mutations (Table 2) based on their involvement in the innate immune response and host defense against microbes. Mutations identified through WES were validated by direct sequence analysis or specific genotyping assays. The mutations were evaluated in an independent cohort of an additional 188 PPROM cases and 175 controls.

**Table 2:** Candidate genes selected for analysis

Category	Gene IDs and (OMIM number)
Innate immune response modulators	*CARD6* (* 609986), *CARD8* (* 609051)
	*IL10* (* 124092), *IL10RA* (* 146933)
	*IL10RB* (* 123889), *NFKBIA* (* 164008)
	*NFKBIB* (* 604495), *NFKBID,*
	*NFKBIE* (* 604548), *NFKBIZ* (* 608004)
	*NLRP3* (* 606416), *NLRP10* (* 609662)
	*NLRP12* (* 609648), *NOD1*(* 605980)
	*NOD2* (* 605956)
	*SOCS1*(* 603597), *SOCS2* (* 605117)
	*SOCS3* (* 604176), *SOCS4* (* 616337)
	*SOCS5* (* 607094), *SOCS6* (* 605118), *TLR10* (* 606270)
LPS detoxification	*ALPP* (* 171800), *AOAH* (* 102593)
Antimicrobial peptides/proteins	*BPI* (* 109195), *CAMP* (* 600474)
	*DEFA1* (* 125220), *DEFB1* (* 602056)
	*DEFB4A* (* 602215), *DEFB103A* (* 606611)
	*FUT2* (**+**182100), *LBP* (* 151990),
	*LTF* (* 150210), *LYZ* (* 153450)
	*MBL2* (* 154545)

### Mutations in genes negatively regulating innate immunity

We detected mutations in the *CARD6, CARD8, NLRP10, NLRP12, NOD2,* and *TLR10* genes (Table 3). Several of these were only found in PPROM cases (*CARD6, NLRP10,* and *NOD2*) in both WES and the follow‐up genotyping cohorts. The SNP for the *CARD6* nonsense mutation has two alternative alleles C or G. We confirmed by DNA sequence analysis that the PPROM case had the G allele creating the stop codon TAG, which truncates the 1037 amino acid protein at position 560, which retains the caspase activation and recruitment (CARD) domain, but deletes the IMPDH (inosine 5′‐monophosphate dehydrogenase/GMP reductase) domain and C‐terminal proline‐rich domain. This nonsense mutation was detected in 2 PPROM cases (combined WES and follow‐up genotyping) and none of the combined term pregnancy controls. The one heterozygous *NLRP10* nonsense mutation detected only in a PPROM case truncates the 655 amino acid protein at position 103. The *NOD2* frameshift mutation truncates the C‐terminal 33 amino acids from the 1040 amino acid protein, disrupting a leucine‐rich repeat. Mutations in *CARD8, NLRP12* and *TLR10* were found in both PPROM cases and controls.

**Table 3:** Damaging mutations identified in genes involved in modulation of the innate immune response in PPROM cases

Gene ID	Chromosome	Position	SNP ID	Ref‐, allele	Alternate‐, allele	Effect	Minor allele	AA position, (residue change)c	Minor allele, frequency cases/controls	Sequence variant
*AOAH*	7p14.2	36514524	rs145455591	C	T	Nonsense	T	556	0.036/0.026	NC_000007.14:g.36514524C>T
*CARD6*	5p13.1	40853011	rs150487186	T	G	Nonsense	G	560	0.004/0.000	NC_000005.10:g.40853011T>G
*CARD8*	19q13.33	48231760	rs140826611	‐	AA	Frameshift	AA	148	0.016/0.006	NC_000019.10:g.48231760_48231761insAA
*DEFB1*	8p23.1	6870777	rs5743490	C	A	Nonsense	A	37	0.011/0.000	NC_000008.11:g.6870777C>A
*FUT2*	19q13.3	48703417	rs601338	G	A	Nonsense	A	154	0.374/0.376	NC_000019.10:g.48703417G>A
*FUT2*	19q13.3	48703041	rs143482452	C	T	Nonsense	T	29	0.002/0.000	NC_000019.10:g.48703041C>T
*FUT2*	19q13.3	48703767	rs1799761	C	‐	Frameshift	C	271	0.007/0.012	NC_000019.10:g.48703767delC
*MBL2*	10q21.1	52768256	rs74754826	G	T	Nonsense	T	210	0.011/0.000	NC_000010.11:g.52768256G>T
*NLRP10*	11p15.4	7961305	rs765522475	C	T	Nonsense	T	103	0.002/0.000	NC_000011.10:g.7961305C>T
*NLRP12*	19q13.42	53795911	rs35064500	C	T	Nonsense	T	1017	0.021/0.007	NC_000019.10:g.53795911C>T
*NLRP12*	19q13.42	53795917	rs776426826	AG	‐	Frameshift	‐	1015	0.002/0.003	NC_000019.10:g.53795917_53795918delAG
*NOD2*	16q12.1	50729867	rs2066847	‐	C	Frameshift	C	1007	0.004/0.000	NC_000016.10:g.50729867_50729868insC
*TLR10*	4p14	38774483	rs62617795	C	T	Nonsense	T	370	0.020/0.016	NC_000004.12:g.38774483C>T
*TLR10*	4p14	38775590	rs140873456	A	G	Start loss	G	1	0.003/0.003	NC_000004.12:g.38775590A>G

Mutations identified through WES (76 PPROM, 43 term controls) were validated by direct sequence analysis or genotyping using TaqMan reagents. The mutations were evaluated in an independent cohort of an additional 188 PPROM cases and 175 controls. Genotyping was performed on the Agena MassArray iPLEX platform. All allele frequencies were based on called genotypes excluding missing samples or those samples without a genotype call. MAF, minor allele frequency.

### Mutations in LPS detoxifying enzymes

A nonsense mutation was found in *AOAH*, which encodes an enzyme that catalyzes the hydrolysis of acyloxylacyl‐linked fatty acyl chains from LPS. The nonsense mutation disrupts the 688 amino acid protein at position 556, retaining the lipase consensus sequence. This mutation was found in both PPROM cases and term controls.

### Mutations in antimicrobial protein genes

A heterozygous nonsense mutation was found in *DEFB1*, which encodes beta‐defensin 1, an antimicrobial factor that is produced by amnion epithelial cells. The rs5743490 SNP reference allele is C with two reported alternatives: T, which results in a synonymous codon change that is functionally not significant, and A which creates a stop codon (TGA). We sequence verified that the allele in our PPROM cases was an A. This stop codon truncates the mature beta defensin 1 peptide sequence after four amino acids, so no active peptide is made (Porto et al. 2016). Additionally, the translated truncated N‐terminal peptide could serve as a dominant negative, competing for the intact signal peptide or processing protease of intact beta‐defensin 1 peptide encoded by the other *DEFB1* allele. The heterozygous *DEFB1* mutation was found in 6 PPROM cases (WES and follow‐up genotyping combined) and no term controls.

A heterozygous nonsense mutation in *MBL2* was identified which deletes the 38 terminal amino acids in the C‐type lectin carbohydrate recognition domain of the 248 amino acid protein. The reference allele of this SNP is a G, with alternate alleles of C, producing a benign missense variant or a T, which creates a TAG stop codon. We confirmed by DNA sequence analysis that the minor allele in our PPROM cases was a T. This nonsense mutation was detected in six of the total PPROM cases and none of the total term controls. Using RT‐PCR, we demonstrated that the *MBL2* gene is expressed in fetal membranes (Fig. S1).

Three mutations were discovered in the *FUT2* gene, which encodes a fucosyltransferase involved in protecting epithelium from bacterial infection. One of the nonsense mutations (rs143482452) was found in one PPROM case (combined WES and follow‐up genotyping cohort) only, and not in the combined term controls. Another one (rs601338) has a relatively high minor allele frequency and was detected in PPROM cases and term controls. The *FUT2* gene is expressed in amnion epithelial cells, and mutations that disrupt the protein cause the “nonsecretor” phenotype, which is associated with absent ABH blood groups (Goto et al. 2016).

All of the mutations described above were heterozygous, except for *FUT2* rs601338. In the case of this common mutation, there were 16 homozygous PPROM cases (21%) of the 76 cases, and four homozygous controls (9.3%) out of the 43 term pregnancies. Among this cohort, seven subjects had di‐genic mutations, two with the *TLR10* rs62617795 mutation and the *CARD8* mutation; two with *AOAH* mutations, one with a *TLR10* rs62617795 mutation, and one with the *CARD8* mutation; and three with the *FUT2* rs601338 mutation in combination with either the CARD6 mutation, *MBL2* mutation, and *NLRP12* nonsense mutation.

We found no nonsense or damaging frameshift mutations in *ALPP, BPI, CAMP, DEFA1, DEFB4A, DEFB103A, IL10, IL10RA, IL10RB, LBP, LTF, LYZ, NLRP3, NFKBIA, NFKBIB, NFKBID, NFKBIE, NFKBIZ, NOD1, SOCS1, SOCS2, SOCS3, SOCS4, SOCS5*, and *SOCS6*. Therefore, these genes did not undergo further interrogation.

Of the 14 mutations identified through WES, 10 had minor allele frequencies in the combined WES and follow‐up genotyping cohort that were nominally greater in PPROM cases than term controls. The allele frequency of two mutant alleles were similar in cases and controls, and two mutations were more frequent in controls than PPROM cases. A paired Wilcoxon rank sum test estimated that across loci, variants were overrepresented in PPROM cases compared to term controls (Empirical *P*‐value from 10K permutations = 0.0416).

In addition to nonsense and damaging frameshift mutations, a number of rare predicted damaging or known pathogenic missense mutations (e.g., *NOD2* rs34936594) were identified through WES in the candidate genes (Table S3). The allele frequencies of these missense mutations were higher in the 76 PPROM cases than the 43 term controls. The association of these predicted rare missense variants with PPROM needs to be replicated with a larger sample size.

## Discussion

Our working hypothesis of whether neonatal genes that negatively regulate innate immunity or help the host combat microbes and their noxious products would be more likely to harbor rare, damaging mutations in PPROM cases was supported by our findings. Interestingly, there were a number of important negative regulators of innate immunity and the host defense system that were not mutated (e.g., *IL10, IL10RA, IL10RB NLRP3, SOCS1, SOCS2, SOCS3, SOCS4, SOCS5, SOCS6, NFKBIA, NFKBIB, NFKBID, NFKBIE, NFKBIZ,* and *NOD1*). Of course, the limited WES sample size may have precluded the detection of very rare alleles in these genes.

Inflammasomes and toll‐like receptors are critical to host defense mechanisms during the physiological and pathological inflammatory processes in the chorioamniotic membranes that accompany labor. Thus, it is not unexpected that mutations in genes that negatively regulate the inflammasome as well as the toll‐like receptors were detected in PPROM cases (Gotsch et al. 2008; Eisenbarth et al. 2012; Oosting et al. 2014).

Mutations in genes encoding host defense mechanisms against microbes had been anticipated based on studies documenting differential expression of the proteins in fetal membranes associated with labor with ruptured and nonruptured membranes (Erez et al. 2009) Notable in this regard are the rare heterozygous damaging mutations in *DEFB1*,* FUT2* and *MBL2* that were found only in PPROM cases. Variation in these genes have been previously associated with increased risk of infection and in some cases preterm birth (Annells et al. 2005; Gibson et al. 2011; Jaffe et al. 2013).

The discovery of a rare nonsense mutation in the *DEFB1* gene is of interest in that variation in this gene (rs1047031, a SNP in the 3′‐UTR) has been associated with chronic and aggressive periodontitis, a condition associated with preterm birth (Schaefer et al. 2010). However, the functional significance of the rs1047031 minor allele has not been established.

Polymorphisms in the *MBL2* gene are more frequent in African‐Americans and multiple studies have suggested an association between *MBL2* genetic variants that result in diminished MBL2 protein levels and preterm birth, and conditions commonly found in preterm pregnancies including chorioamnionitis (Annells et al. 2004, 2005; Gibson et al. 2011; Jaffe et al. 2013; Capece et al. 2014; Nedovic et al. 2014). Our discovery of a nonsense mutations that significantly truncates the MBL2 protein is thus consistent with the notion that loss of this antimicrobial protein increases risk of prematurity.

Given the distribution of allele frequencies of *FUT2* mutations we identified, we speculate that the “nonsecretor” type is not a strong risk factor for PPROM since the more common mutation was found at allele frequencies that were similar in PPROM cases and controls. It is possible, however, that if both mother and fetus harbor mutations in *FUT2* that there could be an increased risk of PPROM, a possibility that we did not explore.

It is noteworthy that genes associated with inflammatory bowel disease also appear to have an association with PPROM, including *CARD* and *NLRP* genes, *NOD2* and *BRIC2* (Hugot et al. 2001; Andreoletti et al. 2017). Although not included in the 35 candidate genes, a novel heterozygous nonsense mutation in *BIRC2* (NC_000011.10: g.102248476T>G), creating a stop codon at position 539 in this 618 amino acid protein, which deletes the C‐terminal zinc finger domain)*,* a gene that negatively regulates the NOD1/NOD2 signaling pathway, and has been recently found to be associated with pediatric inflammatory bowel disease, was discovered in the WES of one PPROM case and no term controls (Andreoletti et al. 2017). A heterozygous damaging frameshift mutation (rs779381525, NC_000010.10 g.49440248_49440249insA) was detected in *FRMPD2*, another gene associated with the NOD2 pathway, in one WES PPROM case.

Chorioamnionitis is often found in PPROM fetal membrane specimens, and the pathways that lead to an accentuated bowel inflammation in Crohn's disease and ulcerative colitis may also contribute to the severity of chorioamnionitis and therefore risk of PPROM. Preterm birth is associated with maternal inflammatory bowel disease but there are no reports that we are aware of that link inflammatory disease in offspring to increased risk of preterm birth and PPROM (Caruso et al. 2014; Getahun et al. 2014; Palomba et al. 2014; Bröms et al. 2016; Shand et al. 2016).

We previously examined the association between 2936insC (rs2066847) in the *CARD15/NOD2* gene and PPROM in African‐Americans and reported that this frameshift mutation was only found in term controls (Ferrand et al. 2002a). This study used genotyping by restriction length polymorphism (RFLP) with digestion with *Nla* IV which cuts the sequence: GGNNCC. We re‐evaluated the putative mutations in the control samples previously analyzed using DNA sequencing and discovered that none of them harbored the frameshift mutation, indicating that the RFLP genotyping was flawed. The genotyping methods employed in the present study can distinguish the frameshift mutation, and therefore provides evidence that 2936insC is a risk allele for PPROM.

The mutations that we identified could be spontaneous, or inherited from the father or mother (Li et al. 2017). We speculate that maternal inheritance may be most likely in the setting of PPROM, since an enhanced maternal reproductive tract inflammatory response to bacteria or viruses, or deficiency in endogenous antimicrobial defenses would presumably act in synergy with similar defects in the fetus when both mother and fetus are heterozygous for damaging mutations (Plunkett et al. 2009).

In conclusion, our WES studies, supplemented with additional target genotyping, revealed a number of rare damaging mutations, the majority being heterozygous, that are more frequent in neonates born of pregnancies complicated by PPROM. These findings suggest that the increased risk of preterm birth in African‐Americans may be conferred by mutations and damaging missense variants in genes encoding proteins involved in dampening the innate immune response and protecting the host against microbial infection.

## Conflict of Interest

The authors have no conflicts of interest to declare.

## Supporting information


**Figure S1.** *MBL2* mRNA expression in fetal membrane samples from normal term pregnancy.
**Table S1.** Primers used for mutation verification by DNA sequence analysis.
**Table S2.** iPLEX genotyping design.
**Table S3.** Predicted damaging SNPs in innate immunity genes.
