# Supplementary material for: Mutations in fetal genes involved in innate immunity and host defense against microbes increase risk of preterm premature rupture of membranes (PPROM)
Source: Mol Genet Genomic Med. 2017 Aug 23;5(6):720–9. doi: 10.1002/mgg3.330 (PMC5702565; doi:10.1002/mgg3.330)
Supplement: Supplementary file 1 — Figure S1. MBL2 mRNA expression in fetal membrane samples from normal term pregnancy. Table S1. Primers used for mutation verification by DNA sequence analysis. Table S2. iPLEX genotyping design. Table S3. Predicted damaging SNPs in innate immunity genes. [file MGG3-5-720-s001.docx]

| GENE | PRIMERS |
| --- | --- |
| *MBL2* | F 5’-GCC ACC CCC AGG AAT GCT G-3’  R 5’-GAG CAG GGG ACG TCA TTC CA-3’ |
| *BIRC2* | F 5’-CGG AAG AAC AGA ATG GCT CTC T-3’  R 5’-CGC ATC TGG CCA GTC CTA AGA A-3’ |
| *NLRP12* | F 5’-AGA AAG AAC TGG TCA TCA TCC C-3’  R 5’-GCC TCC GAA TAA CAT AAG TAG C-3’ |
| *DEFB1* | F 5’-GCT TTC GCG AGA TGT TCT CAA A-3’  R 5’-AGT TCA TTT CAC TTC TGC GTC A-3’ |

**Supplemental Table 1. Primers used for mutation verification by DNA sequence analysis**

*FRMPD2* F 5’- AATGAGACAATGTGTGTAAAGTG-3’

R 5'- TGCCCAAGAGATGCTTACTTTTC-3'

**Supplemental Table 2. iPLEX Genotyping Design**

**rs765522475:** TCCTCTAGGCAGCGCACATGCTCTC**[**A/G**]**GTATACTTCTCTGTAATCTGAGCCA

**rs776426826:** AGCCGCTTGCAAAGCAGTCGGACAC**[**-/CT**]**GTGTCCCCTAGGGCGTTGTTGGTCA

**rs2066847:** CCAATAACTGCATCACCTACCTAGGGGCAGAAGCCCTCCTGCAGG**[**-/C**]**CCCTTGAAAGGAATGACACCATCCTGGAAGTCTGGTAAGGCCCCT

**rs150487186:N**TTTGATGGAA**N**TTTCTTCAGCTGTG**N**TTTTTTTCACTGACTGTT**[**C/G/T**]**AGGTGAGAAGGAATGGGACTTGCTAATGTTTTTAGGAGAGGCT

**rs140873456:**TCATAACAATACTACAAAATATGTAAATGTTTCTGATGAGTCTCA**[**C/T**]**TGTATTTCCAAGGATTTTACCACTTATTTCCTCATATGAATGATG

**rs140826611:** CAGAAACTGACGATTTTTATAATCTTCTTCGATCTCAAAACAGAC**[**-/TT**]**TTAGAAGCATAAGAGGAAACTATTTGATTCTCTTCTGAGCAAAT

**rs62617795:**CCAGTTTATTGCCATTCAAAATGAGAGTTTTCAAGTGAGGCAGTT**[**A/G**]**GATAGTTCTTTTAAACAACTCGTCTGTTAAGATATTATTGGCAAA

**rs145455591**:TTCTGCTGTCGCATGTCAGGTTTCCCTTTATCCATGATGTTAGCT**[**A/G**]**GGTCCATTCTTGGTTGTCCAGTGTCTGGCTTGTCATTAATATGCC

**rs143482452:**CA**N**TTCA**N**CC**N**C**NN**TGTCTTTA**NN**G**N**TTCC**N**CT**N**TATTTCA**NN**TT**[**C/T**]**AGCAG**NN**GCTA**NNN**AA**N**ATTCAA**N**CCA**N**GTGGGAGTTAC**NN**GT**N**C

**rs1799761**: TCCCA**NNN**TGAT**NN**GGTGTTTGCTGG**N**GATGGCA**N**TGA**N**GGCT**N**A**[**-/C**]**CTGCCAAAGA**N**TTTG**N**TCTA**N**T**N**ACACAGTGTAA**N**CACA**N**CATCA

**rs35064500:**CTCGGAGTTTGCAGCCAGGATGGCTCAGCCGCTTGCAAAGCAGTC**[**A/G**]**GACACCTGTGTCCCCTAGGGCGTTGTTGGTCAGGTAAAGGTCGGT

**rs5743490**:TAAC**NN**T**N**TC**NN**A**N**G**N**C**N**TGG**N**C**N**CAG**NNNNN**AT**NN**TT**N**CAATTG**[**A/C/T**] NN**C**NN**C**N**GTGGA**NN**GCAA**NNN**CT**N**T**N**TT**NN**G**N**CT**N**CC**NN**A**NNN**

**rs601338**:AC**N**TCC**NN**GGGGA**N**TAC**N**TC**NN**CTTCAC**NN**GCTA**N**CCCTGCTCCT**[**A/G**]N**ACCTTCTACCA**NN**A**N**CTC**NN**CCA**N**GAGATCCTC**N**AG**N**AGTTCAC

**rs74754826:**G**N**A**N**TACACA**NN**CTT**N**ATCAGAACCAGCATTGTTGGGTTCACCCT**[**A/C/T**]**GTTCCAGTTTGTGTAGGTCAGTCTATTTCCTGTCAGATCCACA

**Supplemental Table 3. Predicted Damaging SNPs in Innate Immunity Genes**

**Predicted Damaging SNPs in Innate Immunity**

| **Gene** | **SNP** | **MAF in WES Cases/Controls** | **Chromosome Position** | **Sequence Variant** | **Functional Consequence** |
| --- | --- | --- | --- | --- | --- |
| *CARD6* | rs146689225 | 0.007/0.000 | 40852929 | NC_000005.10:g.40852929C>T | Missense |
| *CARD8* | rs34632751 | 0.007/0.000 | 48211939 | NC_000019.10:g.48211939T>C | Missense |
| *MBL2* | rs5030737 | 0.007/0.000 | 52771482 | NC_000010.11:g.52771482G>A | Missense |
| *NLRP10* | rs765522475 | 0.007/0.000 | 7961305 | NC_000011.10:g.7961305G>A | Nonsense |
| *NLRP12* | rs374428779 | 0.007/0.000 | 53824137 | NC_000019.10:g.53824137A>C | Missense |
| *NOD2* | rs34936594 | 0.007/0.000 | 50699655 | NC_000016.10:g.50699655C>G | Missense |
| *NOD2* | rs140716236 | 0.013/0.000 | 50711088 | NC_000016.10:g.50711088C>T | Missense |
| *NOD2* | rs5743277 | 0.007/0.000 | 50712018 | NC_000016.10:g.50712018C>T | Missense |
| *NOD2*  *TLR10* | rs5743272  rs114573525 | 0.013/0.000  0.007/0.000 | 50710966  38775211 | NC_000016.10:g.50710966A>G  NC_000004.12:g.38775211T>C | Missense  Missense |

Missense variants, predicted to be damaging by both Polyphen2 (HumDiv - probably damaging) as well as SIFT (damaging) platforms, were detected by WES (76 PPROM case, 43 term controls) and verified by genotyping with an alternative method as described in the legend to Table 3. MAF=minor allele frequency

RT-PCR: Total RNA was isolated with TRIzol® (Life Technologies, Inc., Grand Island, N.Y.) from normal fetal membranes. The RNA was reverse transcribed with RETROscript kit (Ambion, Austin, TX) according to the manufacturer’s instructions, and the cDNA was amplified with primers specific for *MBL2* gene. Fwd primer 5’-GCCACCCCCAGGAATGCTG-3’ and reverse primer 5’-GAGCAGGGGACGTCATTCCA-3’. Amplification product was resolved on 1% agarose gels stained with ethidium bromide.


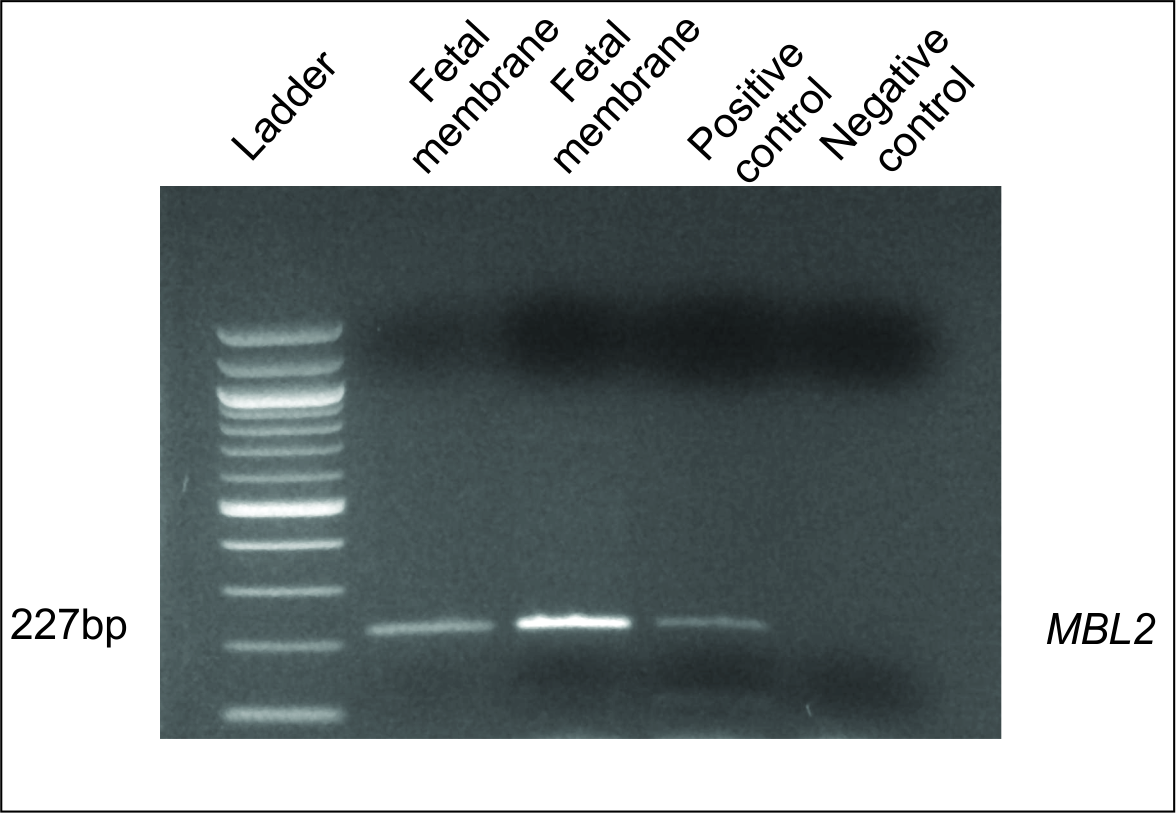


**Supplementary Fig 1.** ***MBL2* mRNA expression in fetal membrane samples from normal term pregnancy.** RT-PCR gel image for *MBL2* (227 bp) using RNA from fetal membrane samples obtained from normal term pregnancy (gestational age > 37 weeks) line 2 and 3. RNA from normal placenta was used as a positive control (lane 4). The *MBL2* cDNA amplicon was sequence- verified.
